# Prevalence of zoonotic helminth infections in ruminants slaughtered at Haromaya Municipal Abattoir in Eastern Ethiopia

**DOI:** 10.1016/j.onehlt.2025.101094

**Published:** 2025-05-29

**Authors:** Isayas Asefa Kebede, Gelan Dule Dahesa, Endrias Zewdu Gebremedhin

**Affiliations:** a School of Veterinary Medicine, Ambo University, P.O. Box 19, Guder, Ethiopia; b School of Veterinary Medicine, Wolaita Sodo University, Wolaita Sodo, Ethiopia; c Department of Animal Health, Oromia Job Creation and Vocational Bureau of Gedo TVET College, Gedo, Ethiopia; d Ethiopian Veterinary Association, P. O. Box 2462, Addis Ababa, Ethiopia

**Keywords:** Haromaya, Helminth, Ruminants, Zoonotic

## Abstract

Zoonotic helminth parasites are naturally transmitted between animals and humans and have public health importance. A cross-sectional study was carried out from November 2023 to July 2024. This study aimed to estimate the prevalence, associated risk factors, and economic impact of zoonotic helminth infections in ruminants slaughtered at Haromaya Municipal Abattoirs, Eastern Ethiopia. A simple random sampling technique was used to select the animals used in this study. The prevalence was determined based on records of parasitic infections identified during postmortem examinations of 400 animals (245 cattle, 86 goats, and 69 sheep). The overall prevalence of zoonotic helminth parasites was 52.3 % (95 % CI: 25.2–70.5). The most prevalent parasitic infection was *Fasciola* species infections (30.3 %), and the lowest was *cysticercosis bovis* infection (4.0 %). Out of the 400 livers inspected, 30.0 % tested positive for *Fasciola* species, with *F. hepatica* accounting for 15.5 %. An overall prevalence of 3.8 % for *C. bovis* was recorded, with 2.5 % in the masseter muscle. Among the 111 cysts studied, there were 62 fertile and 49 non-fertile cysts. In the study area, males had a significantly higher prevalence of helminth infections (62.8 %) than females (40.5 %), with males being 2.5 times more likely to be infected (OR = 2.5; 95 % CI: 1.7–3.7; *p* < 0.05). Annual direct financial losses from zoonotic helminth infections were estimated at 98,363,520 ETB (around 786,908.16 USD), underscoring their widespread and significant economic impact in the study area. Therefore, enhancing sanitary conditions, routine meat inspection, and reporting systems in abattoirs are encouraged.

## Highlights

• Zoonotic helminths were found in 52.3 % of ruminants at Haromaya Abattoirs, with fasciolosis most common (30.3 %).
• Males had a higher infection rate (63.2 %) than females (36.8 %) and were 2.5 times more likely to be infected.
• Annual losses from condemned organs were about 98.4 million ETB (786,908 USD).

## 1 Introduction

Zoonotic helminth parasites are infections that naturally spread between vertebrate animals and humans and vice versa. They threaten public health, particularly in developing countries [1,2]. Emerging and endemic zoonotic diseases threaten not only the health of animals and humans but also global health security. Zoonotic infections account for around 60 % of recognized infectious diseases and up to 75 % of new or emerging infectious diseases [3]. Many major ruminant-borne zoonotic parasite ailments exist, including fasciolosis, cysticercosis, and hydatidosis [4,5]. Bovine parasitic zoonoses are prevalent and have posed a risk to public health in developing countries where raw meat consumption and inappropriate disposal of condemned carcasses are common [2].

Helminth infections like fasciolosis, cysticercosis, and hydatidosis pose major public health and economic challenges [[6], [7], [8]]. Fasciolosis is caused by *Fasciola hepatica* and *F. gigantica* [9]. It causes substantial economic losses in the livestock sector, leading to liver condemnation and compromised immunity [7]. While *F. hepatica* is found above 1800 masl, *F. gigantica* is more common below 1200 masl [9,10]. The disease presents in acute, sub-acute, and chronic forms in animals, causing anemia, weight loss, and eventually death, with diagnosis based on clinical signs, ecology, and laboratory or postmortem confirmation [8,[11], [12], [13]].

*Taenia saginata* is a two-host parasite, involves cattle as intermediate (*Cysticercus bovis*) and humans as definitive hosts. Cattle get infected by ingesting eggs from human feaces, and humans become infected by consuming raw or undercooked beef [6]. Though cattle often show no signs, heavy infections may cause myocarditis [6,14]. Meat inspection focuses on muscles like the external and internal masseter and pterygoid muscles, heart, tongue, diaphragm, and esophagus, but has low sensitivity, often missing infections [15]. In humans, infections may go unnoticed or cause nonspecific gastrointestinal symptoms, with serious complications possible in rare cases [16]. The disease contributes to significant financial loss and public health risks [6,7].

Hydatidosis is caused by *Echinococcus granulosus*, and is another major zoonotic parasite that disrupts organ function and reduces productivity in livestock production [7]. It behaves like cancer, damaging organs and leading to losses in meat, milk, and contributing to economic loss as well [4,7,9]. Diagnosis relies on visual inspection, palpation, and incision during meat examination [17].

Zoonoses caused by parasites are a diverse category of infections with variable host ranges and transmission mechanisms [11]. Human and environmental factors influence their distribution, prevalence, and transmission patterns [9]. Human activities such as deforestation, urbanization, agricultural expansion, and improper waste disposal create favorable conditions for parasite transmission by altering ecosystems and increasing human-animal interactions [9]. Additionally, poor sanitation, lack of clean water, and inadequate healthcare infrastructure contribute to the persistence of zoonotic parasitic infections in certain regions [18]. Climate change further affects the distribution of zoonotic parasites by influencing vector populations, temperature, and humidity, which are critical factors for parasite survival and transmission [18,19].

The economic and public health implications of such zoonoses necessitate effective surveillance to gather enough information to assist in the design and implementation of management methods [5]. Fasciolosis causes an estimated 43,024.458 USD in annual losses in Wolaita Sodo Municipal Abattoir, Ethiopia [20], which is located in the mid-highland zone (1600–2100 masl) [21], similar to Haromaya [22]. The direct economic loss detected in cattle due to liver condemnation by fasciolosis at Hawassa municipal abattoir was estimated to be 291,635.00 USD per year [23]. Other than that, the annual financial loss assessed from organ condemnation due to hydatidosis in southern Ethiopia was estimated to be USD 58,114.62 [24]. Furthermore, Engdaw et al. [25] estimated that the annual economic loss owing to bovine cysticercosis at the Kombolcha meat factories from organ condemnation and carcass weight loss was 73,652.44 USD.

Meat inspection plays a vital role in controlling zoonotic helminth diseases, which pose significant health risks to both humans and animals. In the current study area, the common practice of consuming raw meat (*Kurt* and *Kitfo*) increases the likelihood of zoonotic helminth transmission, yet there are limited researches on its direct impact. Additionally, helminth infections in ruminants lead to organ condemnation, causing financial losses to farmers and the meat industry, but the extent of these losses in Haromaya Municipal Abattoirs remains unclear. The effectiveness of current meat inspection practices in detecting and preventing these infections also requires further evaluation. Moreover, public awareness among consumers, butchers, and abattoir workers regarding zoonotic helminths and prevention measures is lacking. This study aims to address these gaps by assessing zoonotic parasite prevalence, identifying risk factors, evaluating financial losses, and providing recommendations for improved meat inspection and public health interventions in the Eastern Hararghe zone of Oromia, Ethiopia.

## 2 Materials and methods

### 2.1 Study area

**Fig. 1:**
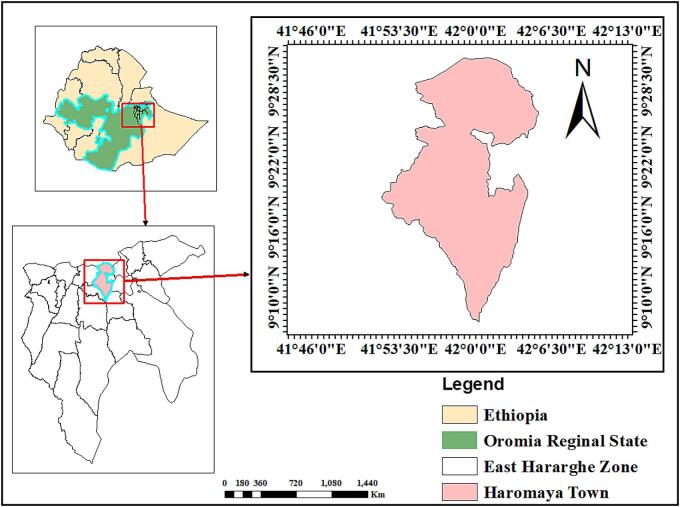
Map of the study area (ArcGIS, 2025).

The study was conducted in Haromaya town, Eastern Ethiopia (Fig. 1). Haromaya is located 510 km East of Addis Ababa on the main road to Harar. The elevation in the region varies between 1600 and 2100 masl. The area has a bimodal rainfall pattern, with short rains from February to May and more prolonged rains from June to September, followed by a dry season from October to February. The average annual rainfall is 492 mm, with a range of 118 to 866 mm. The average maximum and minimum temperatures are 17.0 °C and 9.0 °C, respectively. The ruminant population in *Haromaya* City was estimated to be 79,446 cattle, 28,359 goats, and 18,930 sheep [22].

### 2.2 Study design

A cross-sectional study was conducted at Haromaya Municipal Abattoirs in the Eastern Hararghe zone of Oromia, Ethiopia, from November 2023 to July 2024 to assess the prevalence of zoonotic helminth infections and related risk factors in ruminants, as well as the financial losses caused by infected organ condemnations.

### 2.3 Study animals

The study animals were cattle, sheep, and goats brought to the Haromaya Municipal Abattoir for slaughter and routine meat inspection. The study animals were obtained from Haromaya and the adjacent districts by merchants. During the antemortem inspection, the animals' age, sex, origin, and body condition score were assessed. Cattle were divided into two age groups: adult (≤6 years) and old (>6 years) [23]. Animal body conditions are also classified as medium or good for cattle [26].

Sheep and goats were divided into two age groups: adult (no full permanent teeth) and old [27]. Similarly, the body condition score (BCS) was classified into two categories: medium (score 3) and fat (scores 4 and 5), as no animals were recorded with a poor BCS (scores 1 and 2) [8]. Breeds such as local, crossbreed (local x Holstein Friesian (HF)), and HF were slaughtered in the study area.

### 2.4 Sampling method and sample size determination

The desired sample size was calculated [28], with a 95 % confidence interval and 5 % absolute precision.

$$\frac{Z^{2}\times P_{\text{exp}}\left(1-P_{\text{exp}}\right)}{d^{2}}$$

Where: n = required sample size; Pexp = expected prevalence (*P* = 50 %); d = desired absolute precision. Z = 1.96 for a 95 % confidence interval. Accordingly, the minimum sample size was 384 ruminants. However, a total of 400 animals were randomly selected for this study.

### 2.5 Abattoir survey

#### 2.5.1 Antemortem examinations

All sampled animals underwent antemortem examinations, and only those deemed healthy were selected for slaughter. During the antemortem examination, the sex, age, breed, origin, and BCS of each animal were carefully evaluated and documented [8,27].

#### 2.5.2 Postmortem examinations

After slaughter, carcasses were examined for *Cysticercus bovis* using post-mortem procedures [14,15,25], which involved visual inspection, palpation, and incisions of heart, tongue, triceps, masseter, and neck muscles, with findings recorded according to each organ examined.

Palpation, repeated incisions, and inspection of the bile duct were used during postmortem examinations, as well as the observation of irregularities in liver morphology, and finally, *Fasciola* spp. were examined for their presence [17,20,21].

During postmortem inspection of the abdominal and thoracic organs, the liver, lungs, heart, kidneys, spleen, and others were examined for hydatid cysts. Cysts found in the liver and lungs were carefully removed and visually assessed for their shape [5].

### 2.6 Examination of cysts and viability of protoscolex of *Echinococcus granulosus*

Cysts were collected and taken to the laboratory for examination. Each cyst was checked for deterioration, calcification, fertility, and viability. The cyst wall was incised, and the contents were emptied into a petri dish. Using ×40 magnification, protoscoleces were identified as white dots on the germinal layer, brood capsule, or hydatid sands. Fertile cysts showed viable protoscoleces, which were further tested by mixing with 0.1 % eosin solution; viable ones excluded the dye, while nonviable ones absorbed it [14,29]. Infertile cysts were classified as sterile or calcified. Sterile cysts had a smooth inner lining with mildly turbid fluid, while calcified ones produced a scratchy sound when cut [30]. All results were documented.

### 2.7 *Fasciola* species identification

*Fasciola* were collected in screw-cupped bottles following systematic incisions in the bile ducts and liver parenchyma, and morphological identification using a stereomicroscope [17,31]. Finally, the results were documented.

### 2.8 *Cysticercus bovis* identification

The cyst discovered during carcass inspection was excised along with the surrounding tissue and sent to the laboratory for viability testing. The vitality of the cyst was tested by immersing it in a 40 % ox-bile solution diluted in normal saline and incubating it at 37 °C for 1 to 2 h. A cyst was considered viable if the scolex invaded throughout the incubation phase. *T. saginata* metacestodes were identified by the size of the *C. bovis*, the existence of a rostellum, and the lack of hooks [32].

### 2.9 Estimation of economic loss due to fasciolosis, cysticercosis, and hydatidosis infection

Only direct financial loss was considered when assessing the disease's economic impact. Direct losses were assessed using condemned organs. To calculate the cost of condemned organs, butchers in Haromaya town were interviewed at random to establish the local market price per organ. The data was then analyzed to determine the average price. According to data acquired from butcheries in the Haromaya sub-city, the average cost of sheep and goat liver and lung was 75 and 45 ETH birrs, respectively. Similarly, the average price for bovine lung, tongue, liver, and muscles in the Haromaya sub-city was 75, 100, 350, 1000 ETH birrs, respectively. The direct loss was thus calculated using the formula of Mequaninit and Mengesha [26]. The annual financial loss incurred as a result of organ condemnation due to zoonotic helminth infections was:

Annual cost of condemned organ=NALx%CONDxCL.

Where, NAL = Average number of cattle slaughtered in Haromaya Municipal Abattoir per year. %COND. = Percentage of organs condemned due to zoonotic helminth infections. CL = Mean cost of one organ in the Haromaya sub-city.

Moreover, the direct yearly economic loss for fasciolosis and hydatidosis was calculated as:

yearly economic loss = (PI1 ⨯TK ⨯ C1) + (PI2 ⨯ TK ⨯ C2) + (PM ⨯ TK ⨯ C3) + (PS ⨯ TK ⨯ C4) + (PT ⨯ TK ⨯ C5) [17,33].

**Table:** 

Symbol	Description	Unit/Value (if applicable)
PI₁	Percentage of liver condemnations (fasciolosis) out of the total examined	%
PI₂	Percentage of lung condemnations (hydatidosis) out of the total examined	%
PM	Percentage of masseter muscle involvement out of the total examined	%
PS	Percentage of shoulder muscle involvement out of the total examined	%
PT	Percentage of tongue involvement out of the total examined	%
C₁	Average market price of liver	ETB
C₂	Average market price of a lung	ETB
C₃	Average market price of the masseter muscle (per kg)	ETB
C₄	Average market price of shoulder muscle (per kg)	ETB
C₅	Average market price of tongue	ETB
TK	Average annual slaughter of ruminants	Number of animals (head/year)

### 2.10 Data management and analysis

Data were entered and recorded in a Microsoft Excel 2016 spreadsheet before being analyzed with STATA^@^ software 14.0 (Stata Corporation, Texas, USA, 2006). The total prevalence was determined by dividing the number of *E. granulosus*, *Fasciola* species, and *C. bovis*-positive animals by the total number of animals examined. Univariable and multivariable logistic regression analyses were used to explore the impact of risk factors (origin, age, sex, animal breed, and BCS) on the outcome variable of co-infection status. Univariable logistic regression was used to assess the risk of zoonotic helminth infections in slaughtered ruminants. Potential risk factors with *p*-values ≤0.25 were analyzed using multivariable logistic regression and backward elimination. Hosmer and Lemeshow statistics, as well as the Receiver Operating Curve (ROC), were used to assess model fit and validity [34]. All cases were considered statistically significant with a *P*-value of <0.05. The financial loss was assessed by the total number of organs condemned owing to *E. granulosus*, *Fasciola* species, and *C. bovis* infections and the total number of animals slaughtered during the study period.

## 3 Results

A total of 400 animals (245 cattle, 86 goats, and 69 sheep) were examined for zoonotic helminth infections, and an overall prevalence was recorded at 52.3 % (95 %CI: 25.2–70.5). The prevalence of *Fasciola* species and *C. bovis* was recorded at 30.3 % and 4.0 %, respectively. Similarly, animals from Haromaya (54.4 %) and those with a medium body condition score (53.4 %) showed the highest rates of zoonotic helminth infections compared to their counterparts. Table 1 shows the prevalence of zoonotic helminth infections according to the risk factors assessed.

**Table 1:** Prevalence of zoonotic helminth infections in ruminants slaughtered at Haromaya Municipal abattoir.

Risk factors	Categories	NAE	NPA	Prevalence	95 %CI
Sex	Female	190	77	40.5	33.7–47.7
	Male	210	132	62.8	56.1–69.2
Age	Adult	298	153	51.3	45.6–57.0
	Old	102	56	54.9	45.1–64.3
BSC	Good	147	74	50.3	42.3–58.4
	Medium	253	135	53.4	47.1–59.5
Breed	Holstein Frisian (HF)	12	4	33.3	12.5–63.7
	Cross-breed	16	8	50	26.6–73.4
	Ogaden sheep	69	24	34.8	24.4–46.8
	Local	303	173	57.1	51.5–62.6
Origin	Aweday	8	0	0.0	–
	Karsa	65	31	47.7	35.9–59.9
	Haromaya	327	178	54.4	48.9–59.8
Species	Ovine	69	24	34.8	24.4–46.8
	Caprine	86	67	77.9	67.8–85.5
	Bovine	245	118	48.2	41.9–54.4

NB: NAE: Number of animals examined; NPA: Number of positive animals; CI: Confidence Interval.

Univariable logistic regression revealed that only sex and species were the risk factors that had a statistically significant (*p* < 0.05) association (Table 2).

**Table 2:** Univariable logistic regression of risk factors and their odds of exposure.

Risk factors	Categories	Prevalence	OR	95 %CI for OR	*p*-value
Sex	Female	40.5	Ref	Ref	Ref
	Male	62.8	2.48	1.66–3.72	<0.0001
Age	Adult	51.3	Ref	Ref	Ref
	Old	54.9	1.15	0.73–1.81	0.53
BSC	Good	35.4	Ref	Ref	Ref
	Medium	64.6	1.13	0.75–1.69	0.56
Breed	HF	1.9	Ref	Ref	Ref
	Cross-breed	3.8	2	0.42–9.42	0.381
	Ogaden sheep	11.4	1.06	0.29–3.91	0.922
	Local	82.8	2.66	0.78–9.03	0.116
Species	Ovine	11.4	Ref	Ref	Ref
	Caprine	32.1	6.61	3.25–13.5	<0.0001
	Bovine	56.5	1.74	0.99–3.03	0.05

NB: CI: Confidence Interval; OR: Odd Ratio.

The multivariable logistic regression model revealed that only sex was statistically significant (*p* < 0.05). The Hosmer-Lemeshow goodness-of-fit test indicated that the model suited the data (χ2 (Chi-Square) = 382.04; Prob > χ2 = 0.7104; AUC = 79.01 %) (Table 3).

**Table 3:** Multivariable logistic regression of risk factors and their odds of exposure.

Risk factors	Categories	Prevalence	OR	95 %CI for OR	*p*-value
Sex	Female	40.5	Ref	Ref	Ref
	Male	62.8	2.5	1.7–3.7	<0.0001

NB: CI: Confidence Interval; OR: Odd Ratio.

In this study, 30.25 % of *Fasciola* spp. were found in the liver, with *F. hepatica* accounting for the largest percentage (15.5 %). Similarly, *C. bovis* had an overall prevalence of 3.8 %. The masseter and shoulder muscle had the highest and least number of cysts among the *C. bovis*-detected organs, with 10 and 1, respectively. Furthermore, 12 (75 %) of the 16 cysts found were alive (viable), while 4 (25 %) were dead (degenerative) cysts (Table 4). Compared to other organs, the lung had the highest frequency of hydatid cysts (11 %). Of the 111 cysts recovered, 62 were fertile and 49 were non-fertile (Table 4).

**Table 4:** Occurrence of *Fasciola* species, *C. bovis*, and *E. granulosus* in ruminants slaughtered at Haromaya Municipal abattoir.

*Fasciola* species (bovine, ovine)			*C. bovis* (bovine)					*E. granulosus* (bovine, caprine, ovine)						
	Frequency	Percentage	Sites (muscles/organs)	Frequency	Percentage	Viable	Non-viable	Sites (Organs)	Frequency	Percentage	Fertile		Non-fertile	
											Viable	Non-viable	Calcified	Sterile
*F. hepatica*	62	15.5	Masseter	10	2.5	7	3	Lung	44	11.0	18	5	2	19
*F. gigantica*	3	0.8	Tongue	5	1.3	4	1	Liver	39	9.8	10	9	1	19
Immature	56	14.0	Shoulder	1	0.03	1	0	Both	28	7.0	12	8	3	5
Total	121	30.3	Total	16	3.8 %	12	4	Total	111	27.8	40	22	6	43

To obtain TK, if 100 cattle are slaughtered per week on average, 400 per month, and 4800 per year (i.e., 100⨯4⨯12 = 4800), then annual economics loss = (22.4⨯75⨯4800) + (25.3⨯350⨯4800) + (4.5 ⨯100 ⨯ 4800) + (0.83 × 1000 ⨯ 4800) + (2 ⨯ 100 ⨯ 4800) = 82,728,000 birrs (Table 5).

**Table 5:** Direct financial loss due to infected organ condemnation of fasciolosis, bovine cysticercosis, and hydatidosis in ruminants slaughtered at Haromaya Municipal abattoir.

Species	NEA	Organ type	TEO	NPO	PIO (%)	AUPO	Total EL loss (ETB)	Total EL loss USD
Bovine	245	Liver	245	62	25.3	350	42,504,000	340,032
		Lung	245	55	22.4	75	8,064,000	64,512
		Tongue	245	5	2.0	100	960,000	7680
		Shoulder	245	2	0.82 (3 kg)	1 kg = 1000	9,600,000	76,800
		Masseter muscle	245	11	4.5 (6 kg)	1 kg = 1000	21,600,000	172,800
Caprine	86	Liver	86	36	41.9	75	12,067,200	96,537.6
		Lung	86	9	10.5	45	1,814,400	14,515.2
Ovine	69	Liver	69	0	0 %	75	0	0
		Lung	69	8	11.6	45	1,753,920	14,031.36
Total							98,363,520	78,6908.16

NB: NEA: Number of Examined animals; TEO: Total examined organs; NPO: Number of positive organs; PIO: Percent involvement of organ; AUPO: Average unit price of organ; EL: Economic Loss; ETB: Ethiopian Birr; (money conversion rate: 1USD = 125 ETB during study period 2024).

Similarly, to obtain the annual economic loss from slaughtered goats, if 80 goats are slaughtered per week on average, 320 per month and 3840 per year (i.e., 80 ⨯ 4 ⨯ 12 = 3840 birrs), then annual economics loss = (PI1 ⨯ TK x C1) + (PI2 ⨯ TK ⨯ C2) = (10.5 ⨯ 45 ⨯ 3840) + (41.9 ⨯ 75 ⨯ 3840) = 13,881,600 birrs (Table 5). Also, to obtain the annual economic loss of slaughtered sheep, if 70 sheep are slaughtered per week on average, 280 per month and 3360 per year (i.e., 70 ⨯ 4 ⨯ 12 = 3360 birrs), then annual economics loss = (pI1 ⨯ TK ⨯ C1) + (pI1 ⨯ TK ⨯ C1) = (0) + (11.6 ⨯ 45 ⨯ 3360) = 1753,920 birrs (Table 5).

Finally, annual economic losses were calculated by adding all species' economic losses (98,363,520 ETB or 786,908.16$) (Table 5).

## 4 Discussion

In this study, the overall prevalence of zoonotic helminth infections was 52.3 % (95 % CI: 25.2–70.5), and it is consistent with the 35 % prevalence reported in cattle at the El-Minia Governorate Abattoirs in Egypt [5]. However, the current prevalence was greater than the 0.76 % found at an abattoir in Egypt's Mid-Delta [35]. This is attributed to variations in farmers' awareness of parasite prevention and control measures, animal defecation habits in water canals, irrigation of plants and roughages used by animals, and infected water polluted with encysted metacercaria between study areas [36].

In the current study, the most common parasite infection in slaughtered animals was fasciolosis at 30.3 %, which was greater than previous findings of fasciolosis at 0.2 % in Egypt's Mid-Delta [35], 0.58 and 1.4 % in local Egyptian abattoirs [37]. The current finding was consistent with 31.5 % Mecha District, West Gojam Zone [38]. However, it was greater than 24.32 % in Mekelle's municipal abattoir [10]. The disparity in the prevalence of fasciolosis recorded in various studies can be related to a variety of factors, including variances in infection resistance, grazing patterns, and animal breeds, as well as differences in local climatic conditions suitable for intermediate hosts [27].

The current study found that *F. hepatica* had a higher prevalence (15.5 %), followed by *F. gigantica* (0.8 %) and immature (14.0 %). However, this prevalence was lower than previous studies of *F. hepatica* (56.42 %) and mixed infection of *F. hepatica* and *F. gigantica* (5.87 %) [10].

*Cysticercosis bovis* was the least common zoonotic helminth infection in slaughtered animals, accounting for 3.8 % of the total. The result is consistent with 4.24 % at the Bishoftu Municipal Abattoir in Central Ethiopia [14]. However, it was greater than the earlier cysticercosis result of 0.6 % in Egypt's Mid-Delta [35]; however, lower than 7.5 % in Addis Ababa Abattoir, Ethiopia [39] and 18.49 % in northern Ethiopia [15]. The disparity between prior and current prevalence could be attributed to variances in agroclimatic conditions, animal size sampled, human living or management systems, and the area from which the animals arrived. Furthermore, many infections go unnoticed during normal inspection of beef carcasses because cysticerci are readily missed, as they may not be present on routine cuts, given that the majority of cysticercosis cases have light infections [35].

Additionally, observations revealed that, except dead, degenerate, or calcified cysticerci, which typically form white and fibrotic lesions, a careless meat inspector could easily miss a large number of viable cysticerci, which blend in with the pinkish-red color of the meat and are passed on for human consumption [6,7,14]. Variations in meat inspectors' skills and motivation, the pace of slaughter operations, and the quality of meat inspection facilities all contribute to the accuracy of meat inspection. Furthermore, the extent of permissible incisions is limited, as excessive cutting can negatively impact the carcass's market value [39].

Our findings indicated that the masseter muscles (2.5 %), tongue (1.3 %), and shoulder muscles (0.03 %) were the preferred sites for bovine cysticercosis cysts, consistent with previous reports from various endemic regions. However, these results contrast with those of Fesseha and Asefa [14], who reported prevalence rates of 36.6 % for masseter muscles and 35.6 % for the tongue, as well as Kebede et al. [39], who found rates of 28.6 % for masseter muscles, 42.9 % for the tongue, and 14 % for shoulder muscles. The abattoir survey analysis revealed a considerable variance in the anatomical distribution of *C. bovis* in the inspected organs of slaughtered animals. This indicated that the masseter muscles were the first to be identified harboring *C. bovis*. This variance could be attributed to the meat inspector's ability to identify occurrences of parasites, sample size, meat inspection approach, and multiple cuts [14].

In this study, the prevalence of echinococcosis/hydatidosis in ruminants was found to be 27.8 %. This result is similar to previous findings of 22.1 % [40], and 32.1 % [10]. However, it was higher than the 4 % prevalence reported in El-Minia Governorate Abattoirs, Egypt [5]. These disparities in disease prevalence in different places could be attributed to differences in the ecological factors that influence disease occurrence. Different prevalence results may be reported from the same area because of differences in the number of animals examined, the duration, and the months of the study period [5].

The cyst fertility for hydatid cysts examination revealed that 62 of the 111 cysts collected were fertile and 49 were non-fertile. These findings were consistent with findings in cattle slaughtered in the Wolaita Sodo municipal abattoir in southern Ethiopia [17] and the Mekelle Municipal slaughterhouse in Tigray Region [10]. This is because the liver and lungs are the first vast capillary regions that this parasite meets between the portal routes before affecting any other peripheral organ [41]. Out of the 16 cysts of *C. bovis* found, 12 (75 %) were determined to be alive (viable), whereas 4 (25 %) were dead (degenerative). The finding was comparable to the finding of the Bushoftu Municipal abattoir [14].

There was a significant association (*p* < 0.05) between helminth infections and sex. Male animals were about three times more likely than females to have helminth infections (OR = 2.5, 95 %CI: 1.7, 3.7). This finding was consistent with that of abattoirs in all areas of Edo State, Nigeria [42]. The authors of that study hypothesized that the increased infection rate in male cattle could be attributable to the fact that males were more frequently slaughtered for food. At the same time, females were left for milk production and reproduction. This could have prompted the herders to ensure that the females grazed on clean pasture and drank clean water [42].

In this study, the total monetary losses owing to parasite infection, through both organ condemnation and carcass weight reduction, were estimated at 98,363,520 ETB (786,908.16 USD). This economic loss was considerably higher than the 291,635.00 ETB in Hawassa Municipal Abattoir, Southern Ethiopia, and 106,331.3 EGP (16,800.4 USD) in Egypt reported by Yalew et al. [23], and Elmonir et al. [35], respectively. The differences in economic losses between regions were primarily attributed to variations in disease prevalence and local market prices for meat and organs. Infected organs such as the liver, lungs, masseter muscle, shoulder muscle, and tongue were condemned when affected by parasites like *Fasciola* spp., *Echinococcus granulosus*, and *Cysticercus bovis*, rendering them unsuitable for sale and consumption. Moreover, parasitic infections often lead to chronic illness, poor growth rates, and reduced feed efficiency, ultimately resulting in lighter carcasses at slaughter. Since meat is sold by weight, this further contributes to financial losses. Consequently, areas with higher infection rates and better market values naturally experience greater economic impacts from parasitic diseases.

The limitations of this study include the potential for an unrepresentative sample size of the ruminant population in the region, difficulties in tracing the geographical origin of all animals, which may affect the generalizability of the results, variability in inspection procedures, and limited consideration of environmental factors or management practices that could influence zoonoses transmission. Additionally, the cross-sectional nature of the study may not capture seasonal variations in prevalence. A longitudinal study comparing seasonal variations in zoonotic helminth infections, along with an analysis of associated risk factors, would offer a more comprehensive understanding of the disease's prevalence and its dynamics over time.

## 5 Conclusions

The study found that zoonotic helminth infections were common in the study area. *Fasciola* spp.*, Echinococcus granulosus,* and *Cysticercus bovis* were the zoonotic helminths identified in slaughtered animals, with infection significantly associated with sex (*p* < 0.05). The annual direct financial loss sustained owing to parasite infection was estimated to be 98,363,520ETB or 786,908.16 USD. This study revealed a large loss of cheap and dependable protein supplies due to the ineffective use of infected organs or carcasses, stressing the importance of incorporating integrated techniques into disease surveillance and control programs. Thus, an all-encompassing approach focused on improving sanitary conditions and strengthening meat inspection and reporting systems among abattoirs, a routine meat inspection procedure, consistent improvement in veterinary delivery services, including anthelmintic treatment, farmers' awareness creation, and the use of better control measures are encouraged.

## CRediT authorship contribution statement

**Isayas Asefa Kebede:** Writing – review & editing, Visualization, Validation, Supervision, Software, Resources, Methodology, Formal analysis, Data curation, Conceptualization. **Gelan Dule Dahesa:** Writing – original draft, Visualization, Software, Resources, Project administration, Methodology, Investigation, Funding acquisition, Formal analysis, Conceptualization. **Endrias Zewdu Gebremedhin:** Writing – review & editing, Validation, Software, Data curation.

## Consent for publication

Not applicable.

## Ethics approval and consent to participate

Ethical consent was obtained from WSU-RRC (04/03/23). The purpose of the study was well explained to the abattoir officials before taking the samples, and informed consent was obtained to take the appropriate sample through verbal consent.

## Funding

No funding was received for this research.

## Declaration of competing interest

All authors declare no competing conflicts of interest.

## Data Availability

All the datasets generated or analyzed during this study are included in this manuscript.
